# Empowerment, partner’s behaviours and intimate partner physical violence among married women in Uganda

**DOI:** 10.1186/1471-2458-13-1112

**Published:** 2013-12-01

**Authors:** Betty Kwagala, Stephen Ojiambo Wandera, Patricia Ndugga, Allen Kabagenyi

**Affiliations:** 1 Department of Population Studies, School of Statistics and Planning, College of Business and Management Sciences, Makerere University, Kampala, Uganda; 2 Centre for Population and Applied Statistics, Makerere University, Kampala, Uganda

**Keywords:** Intimate partner violence, Decision-making, Alcohol, Controlling behaviours, Uganda

## Abstract

**Background:**

There is dearth of knowledge and research about the role of empowerment, partners’ behaviours and intimate partner physical violence (IPPV) among married women in Uganda. This paper examined the influence of women’s empowerment and partners’ behaviours on IPPV among married women in Uganda.

**Methods:**

The 2011 Uganda Demographic and Health Survey data were used, selecting a weighted sample of 1,307 women in union considered for the domestic violence module. Cross tabulations (chi-square tests) and multivariate logistic regressions were used to identify factors associated with IPPV.

**Results:**

The prevalence of IPPV among women in union in Uganda is still high (41%). Women’s occupation was the only measure of empowerment that was significantly associated with IPPV, where women in professional employment were less likely to experience IPPV. Women from wealthy households were less likely to experience IPPV. IPPV was more likely to be reported by women who had ever had children and witnessed parental IPPV. IPPV was also more likely to be reported by women whose husbands or partners: accused them of unfaithfulness, did not permit them to meet female friends, insisted on knowing their whereabouts and sometimes or often got drunk. Women who were afraid their partners were also more likely to report IPPV.

**Conclusion:**

In the Ugandan context, women’s empowerment as assessed by the UDHS has limited mitigating effect on IPPV in the face of partners’ negative behaviours and history of witnessing parental violence.

## Background

Sexual and gender-based violence is a major public health problem [1,2] with immediate and long term consequences [3]. These include negative psychological or behavioural outcomes, physical injuries, and poor reproductive health outcomes such as heightened risk of HIV and sexually transmitted infections [4,5] gynaecological and sexual disorders, pregnancy complications, miscarriages and low birth weight [6].

High levels of sexual and gender-based violence exist in Uganda. The 2011 Uganda Demographic and Health Survey (UDHS) findings reveal that 27% of women experienced physical violence, and 16% experienced sexual violence within 12 months prior to the survey [7]. Intimate partner physical violence (IPPV) which is a form of Intimate Partner Violence (IPV) is among the most common forms of gender-based violence in Uganda [7]. Among women in union, one in four (25%) experienced physical violence and 21% experienced sexual violence from an intimate partner within 12 months prior to the survey. Overall, 45% of ever-married women had experienced at least one form of violence (emotional, physical or sexual) perpetrated by their current or most recent partner in the past year [7].

IPPV has been linked to gender empowerment. Empowerment is a personal, latent phenomenon and multi-dimensional process [8] that denotes autonomy, power, status, and agency. It entails a process of gaining greater control over one’s life, with ability and freedom to make strategic life choices, control resources and the power to achieve goals [9-11]. Kabeer (2005) relates empowerment to the concept of agency which is the ability of an individual to make and put into effect choices (even in the face of opposition, thus challenging power relations). Exercising of agency in order to realise intended goals is facilitated by access to and control over resources.

Empowerment is usually used with reference to persons who hitherto lack such power, in this case – women [12]. Consideration of the context or broader setting is important [10] since the various aspects of empowerment are usually applied in social contexts and in relation to other persons. It is assumed that women’s empowerment usually results in a better quality of life and in this case, less intimate partner violence. Consequently, economic empowerment of women, for instance is recommended as a protective factor for addressing violence against women by the United Nations [13].

As noted by Simeen et al. (2011), empowerment is often accompanied by responsibilities and sometimes repercussions which could include heightened IPV, neglect or withholding of support. This is particularly the case where putting choices into effect challenges power relations. Some studies have linked women’s economic empowerment to IPPV where economically empowered women had increased likelihood of experiencing IPPV compared to those that were not empowered [13-15]. In India, economic empowerment through earning incomes was found not to be the only protective factor for IPV [13]. According to Dalal (2011), in some cases, violence increases as husbands or partners attempt to compensate for women’s enhanced status and independence due to employment. In Kenya too, imbalances in status between married men and women, where women’s labour force participation and occupational status were higher than the male partners’, increased the risk of violence [14]. This calls for analysis of not only individual/micro aspects but also the broader contexts.

Studies have shown that influence of women’s empowerment could vary with social contexts and outcomes of interest. With reference to fertility goals for instance, Upadhyay and Karasek [16] established that women’s empowerment as assessed on the basis of their participation in household decision making, attitudes towards wife beating, and women’s right to refuse sex was not consistently associated with a desire for smaller families or desired fertility in some Sub-Saharan African countries [14,16,17]*.*

Factors that have been associated with increased risk of IPPV include women’s low education level [18], unemployment, attitudes justifying physical violence, limited decision making autonomy and partners’ behaviours such as excessive drinking [19,20]. With regard to contextual factors, cultures that condone violence, rural residence, poverty/poor wealth status [11] and exposure to war [21] also increased the likelihood of IPPV. In addition, women (and men) who witnessed parental IPV compared to those that did not, were more likely to have supportive attitudes towards IPV and to report IPV victimisation perpetrated by men [17,20]. A study in Ethiopia established that women who witnessed inter-parental violence during childhood, compared to those that did not, were twice more likely to report lifetime IPV and more than one and half times more likely to report current IPV [22].

Controlling behaviours of male partners are a precursor to IPPV. Controlling male partners were more likely to be violent than the less controlling partners according to the WHO multi-country studies of 2006 and 2011 [23,24].

Studies linking gender empowerment and intimate partner physical violence among women in union [10] are yet to be conducted in Uganda. IPPV in this case excludes sexual violence. Although sexual violence is part of physical violence, associated factors could differ and therefore requires separate analysis. This paper assessed the influence of selected aspects of women’s empowerment and male partners’ behaviours on IPPV among women in union in Uganda, controlling for women’s socio-demographic factors.

## Methods

The paper uses data from the 2011 Uganda Demographic and Health Survey (UDHS). The UDHS data were accessed with permission from Measure DHS [25]. This was a cross-sectional nationally representative survey that uses stratified two-stage cluster sampling design [7] based on the sampling frame from the 2002 population and housing census [14]. Detailed description of sampling procedures is reported in the UDHS report [7].

The sample for the domestic violence (DV) module was 2,056 ever-married women. From this sample, we extracted 1,447 women (unweighted sample) in union for further analysis [7] (weighted sample was 1307 women in union). In this paper, women in union included those who were married, living together or cohabiting with their partners. The DV module was based on the shortened and modified version of the Conflict Tactics Scale (CTS) [26]. The survey was carried out based on World Health Organization’s (WHO) ethical and safety recommendations for research on domestic violence [27].

### Measures of outcome variable

In this paper, we operationalized intimate partner physical violence (IPPV) as any physical acts ensuing into abuse by a current or former partner within 12 months prior to the interview [19]. For physical intimate partner violence, women in union were asked the following set of questions: Does/did your (last) (husband/partner) a) Push you, shake you, or throw something at you?; b) Slap you?; c) Punch you with his fist or with something that could hurt you?; d) Kick you, drag you, or beat you up?; e) Try to strangle you or burn you on purpose?; and e) Threaten or attack you with a knife, gun, or any other weapon?

In the UDHS data, a composite variable for less severe (moderate) violence was created from questions a-c above and severe violence from d-f above. In this paper, both severe and moderate IPPV are combined to form a dichotomous measure of IPPV (0 = did not experience IPPV in the last 12 months, 1 = experienced IPPV in the last 12 months). We merged moderate and severe physical violence to come up with an aggregate IPPV. This is based on the observation that IPPV is stigmatized and is therefore usually under reported or toned down.

### Measures of independent variables

In this paper, independent variables are categorised into three: first, women’s empowerment (economic empowerment, participation in decision-making, attitudes justifying physical violence); second, husband/partners’ behaviours (controlling behaviour, attitude towards husband (whether she fears husband/partner) and frequency of getting drunk); and third, contextual factors (socio-demographics) including history of witnessing parental violence as described below. In the rest of the document, “partner” includes husbands. In this paper, measures of empowerment included economic empowerment, participation in decision-making and attitudes justifying physical violence.

Women’s economic empowerment included their occupation and ownership of property (a house). Ownership of a house was recoded into two categories: woman alone/jointly with the partner as the empowered category and partner alone/others as the other. Ownership of a house is included because it is an important and contested asset for women in the Ugandan context.

Participation in household decision making concerning how women’s earning are used, women’s health care, large household purchases, visits to family or relatives and how men’s earnings are used was measured in the DHS. In this paper, we used five measures of decision-making autonomy regarding persons who usually decide: a) how women’s earnings are used; b) on respondent’s healthcare; c) on large household purchases; d) on visits to family or relatives and e) what to do with the money the partner earns. Responses to these questions were re-coded into two categories (1 = woman decides alone/jointly with partner, 0 = partner alone/others).

Attitudes justifying physical violence were measured by questions concerning whether wife beating was justifiable for the following reasons: if the wife a) goes out without telling partner; b) neglects the children; c) argues with the partner and d) refuses to have sex with the partner. Responses to these variables were dichotomous (1 = “yes” or 0 = “no”). Some studies have used composite variables from these measures [19,28]. We opted to use individual rather than composite or aggregate measures of empowerment [19]. The challenge with composite measures is that it is not possible to determine the contribution of specific measures in influencing IPPV. Individual measures may vary with socio-cultural settings. Our interest was to tease out specific measures of empowerment that are significantly associated with violence.

We also considered partners’ behaviour and controlling tendencies as reported by the women. In this category, three measures were used: controlling behaviour, frequency of partner getting drunk and woman’s attitude towards partner. In the UDHS, women in union were asked whether their present partners : a) were jealous if respondents talk with other men; b) accuse respondents of unfaithfulness; c) do not permit respondents to meet female friends; d) tries to limit respondents’ contact with family and e) insist on knowing where respondents were. The responses to these variables were dichotomous (0 = no and 1 = yes). All these control variables were included in the model in order to predict those that significantly predict IPPV. Partner’s frequency of getting drunk was categorised into three groups (0 = never, 1 = sometimes 2 = often). We also included respondent’s attitude towards partner in this category of variables. Women were asked if they were afraid of their partners. This was categorised as 0 = never at all, 1 = sometimes and 2 = most of the times.

Women’s socio-demographic characteristics included: women’s age group, women’s education level, region, place of residence, wealth index, parity or number of children ever born and current marital status (married or cohabiting). The role of witnessing parental IPPV was examined by including a dichotomous measure of whether the respondent reported ever witnessing his or her father beating his or her mother (yes or no).

### Statistical analyses

Frequency distributions were used to describe the characteristics of the respondents. Cross-tabulations were used to investigate associations between IPPV (dependent variable) and women’s empowerment (economic empowerment, attitudes justifying physical violence, decision-making autonomy) and partners’ behaviors and women’s socio-demographic factors. Pearson’s chi-squared (*χ*^2^) tests were used to examine the significant differences between IPPV and independent variables. The level of statistical significance using p-values was set at *p* < 0.05.

Multivariate logistic regression analyses were used to examine the association between IPPV and independent variables whose p-values were less than 0.05 during the chi-square tests. Results are presented in the form of Odds Ratios (OR). The level of statistical significance using p-values was set at *p* < 0.05. All analyses were weighted and performed in STATA version 12.

## Results

### Distribution of respondents by socio-demographic characteristics and measures of empowerment

From Table 1, most (84%) of the respondents were rural residents, had primary education (60%) and 40% were from the richer and richest wealth quintiles. More than half (55%) were married and the rest (45%) were cohabiting. Forty one Percent were aged 25–34 years. A half (50%) of the women had given birth to at least one child in their lifetime.

**Table 1:** Percentage distribution of married women and experience of Intimate Partner Violence (IPPV) in Uganda (DHS 2011)

**Variables**	**% of women**	**% reporting IPPV**	**Frequency**	**p-value**
**Residence**				**0.001**
Urban	16.4	27.8	214	
Rural	83.6	43.6	1093	
**Women’s education level**				**0.002**
No education	17.0	48.5	222	
Primary	60.1	44.2	785	
Secondary	22.9	27.2	299	
**Wealth index**				**0.000**
Poorest	18.6	55.9	243	
Poorer	19.9	48.9	260	
Middle	20.1	47.6	262	
Richer	19.5	32.6	255	
Richest	21.9	22.9	287	
**Current marital status**				**0.046**
Married	55.3	39.9	723	
Cohabiting	44.7	42.5	584	
**Women’s occupation**				**0.000**
Not working	23.7	43.0	310	
Professional	4.0	7.5	52	
Agriculture	53.4	44.3	698	
Sales	18.9	36.6	247	
**Age group**				**0.320**
15-24	29.7	37.2	388	
25-34	40.6	41.6	531	
35+	29.7	44.1	388	
**Region**				**0.000**
Central	28.0	28.5	366	
Eastern	26.3	48.9	344	
Northern	19.2	50.0	251	
Western	26.4	40.0	346	
**Parity**				**0.000**
None	6.7	13.4	87	
1-4	50.0	39.9	653	
5+	43.4	46.7	567	
**Total**	**100.0**	**41.1**	**1307**	
**Woman owns a house alone or jointly**				**0.000**
No	42.7	33.7	558	
Yes	57.3	46.5	749	
**Woman decides alone or jointly on:**				
**Spending her income**				**0.901**
No	56.2	41.2	734	
Yes	43.8	40.8	573	
**Own healthcare**				**0.952**
No	40.8	41.2	533	
Yes	59.2	41.0	774	
**Household purchases**				**0.168**
No	43.8	43.8	572	
Yes	56.2	38.9	735	
**Visits to family or friends**				**0.075**
No	42.4	44.7	555	
Yes	57.6	38.3	752	
**What to do with partner’s income**				**0.543**
No	54.3	42.0	710	
Yes	45.7	39.9	597	
**Beating justified if wife:**				
**Goes out without telling husband**				**0.078**
No	58.9	38.5	765	
Yes	41.1	44.8	535	
**Neglects children**				**0.091**
No	52.1	38.2	680	
Yes	47.9	44.2	624	
**Argues with husband**				**0.000**
No	69.5	36.4	901	
Yes	30.5	52.0	395	
**Refuses to have sex with husband**				**0.029**
No	75.8	38.7	976	
Yes	24.2	47.7	311	
**Burns the food**				**0.954**
No	85.0	41.0	1106	
Yes	15.0	41.3	195	
**Total**	**100.0**	**41.1**	**1307**	

Concerning measures of empowerment, just over half (53%) of the women were engaged in agriculture, more than half (57%) reported ownership of a house either alone or jointly. Participation in decision making among women in union was average. Between four to five women out of ten participated in deciding on: how their earnings were used (44%); their own healthcare (59%); household purchases (56%); visits to family (58%); and how their partners’ earnings were used (46%).

The prevalence of attitudes justifying physical violence varied in proportions. Wife beating was justified if women: went out without telling partners (41%); neglected children (48%); argued with partner (31%); refused sex with partner (24%) and burnt food (15%). Four in ten (41%) of women in union reported experiencing IPPV in the last 12 months preceding the survey. More than half (52%) had witnessed their fathers beat their mothers during childhood.

### Association of IPPV and women’s socio-demographic factors and empowerment

From Table 1, results of the cross tabulations show that all socio-demographic factors with the exception of marital status and age were significantly associated with IPPV (residence, education, wealth index, region, parity).

Economic empowerment factors (women’s occupation and ownership of a house) had significant associations with IPPV. Surprisingly, none of the measures of participation in decision making had a significant association with IPPV among women in Uganda (see Table 1). Measures of attitudes justifying physical violence which were significantly associated with IPPV were: arguing and refusing to have sex with the partner. Witnessing parental violence was significantly associated with IPPV (see Table 1).

### Distribution of respondents by their’ partners’ behaviours

Table 2 presents male partners’ behaviour and controlling tendencies and their association with IPPV. Descriptive results show that spouse/partners’ controlling behaviours was high with regard to being jealous if the respondent talked to other men (56%) and insisting on knowing where the respondent was (55%). Controlling behaviours such as limiting respondents’ contact with family, not permitting the respondent to meet female friends, and accusing the respondent unfaithfulness received less emphasis (18%, 26% and 32% respectively). Nearly half (46%) of the women were afraid of their partners. About four in ten (40%) of the women had partners who got drunk.

**Table 2:** Percentage distribution of women in union by male partners’ behaviour and experience of IPPV in Uganda (DHS 2011)

**Variables**	**% of women**	**% reporting IPPV**	**Frequency**	**p-value**
**Husband/partner jealous if respondent talks with other men**				**0.000**
No	43.6	27.3	570	
Yes	56.4	51.6	737	
**Husband/partner accuses respondent of unfaithfulness**				**0.000**
No	68.1	30.5	890	
Yes	31.9	63.5	418	
**Husband/partner does not permit respondent to meet female friends**				**0.000**
No	73.8	35.2	965	
Yes	26.2	57.6	342	
**Husband/partner tries to limit respondent’s contact with family**				**0.000**
No	81.5	36.7	1066	
Yes	18.5	60.5	241	
**Husband/partner insists on knowing where respondent is**				**0.000**
No	45.2	25.4	591	
Yes	54.8	54.0	716	
**Woman afraid of partner**				**0.000**
Never	54.2	24.4	706	
Sometimes	27.2	58.8	354	
Often	18.6	64.4	242	
**Frequency of getting drunk**				**0.000**
Never	59.8	30.8	782	
Sometimes	15.2	67.5	199	
Often	25.0	49.5	326	
**Respondent’s father ever beat her mother**				**0.000**
No	48.2	29.7	559	
Yes	51.8	52.2	600	
Total	100	41.4	1307	

### Association of IPPV among women in union and partners’ behaviours

All measures of male partners’ controlling behaviour, alcohol consumption and women’s attitudes towards partner were strongly associated with occurrence of IPPV among women in union in Uganda. IPPV was higher among women whose partners accused them of unfaithfulness (64%), limited contact with family (61%), denied them to meet female friends (58%), insisted on knowing women’s whereabouts (54%), and were jealous when they talked to other men (52%). IPPV was also higher among women who were afraid of their partners and whose partners got drunk (see Table 2).

### Multivariate results

Three models were fitted to measure the relationship between IPPV and independent variables. The models excluded variables that were not significant at the bivariate level of analysis. In model 1, IPPV was modelled with attitudes justifying wife beating. The only measure of attitudes towards wife beating that had a significant relationship with IPPV was justification of wife beating if a woman argued with her partner. Women who justified wife beating if a woman argued with her partner had increased odds (OR = 1.87; p < 0.001) of experiencing IPPV in comparison to those that did not.

In model 2, we added partners’ controlling tendencies to the first model. Three variables were significantly associated with IPPV: beating justified when wife argues with partner, partner accuses woman of unfaithfulness and partner insisting on knowing her whereabouts. IPPV was elevated among women who justified wife beating when women argued with their partners (OR = 1.58; p = 0.01); whose partners accused them of unfaithfulness (OR = 2.43; p < 0.001) and whose partners insisted on knowing where they were (OR = 2.00; p < 0.001). In the second model, justification of wife beating if a woman argues with her partner was weakened by controlling tendencies.

In the final model (model 3), socio-demographic variables, women’s attitude towards partner, witnessing of parental IPPV and partner alcohol consumption are added. In this model, attitudes justifying wife beating become insignificant (see Table 3). IPPV was significantly associated with partners’ controlling tendencies, wealth index, occupation, parity, attitude towards partner (whether the woman was afraid of the partner), alcohol consumption and witnessing parental violence as explained below.

**Table 3:** Results of logistic regression of IPPV and empowerment indicators controlling for women’s socio-demographic factors and male partners’ behaviours in Uganda (DHS 2011)

**Experienced intimate partner physical violence (IPPV) in the last 12 months**						
	**Model 1**		**Model 2**		**Model 3**	
	**ORs**	**p-value**	**ORs**	**p-value**	**ORs**	**p-value**
**Beating justified if wife:**						
**Argues with husband**						
Yes	1.871	**0.000**	1.575	**0.012**	1.374	0.142
No (rc)	1.000		1.000		1.000	
**Refuses to have sex with husband**						
Yes	1.066	0.730	1.127	0.527	1.109	0.644
No (rc)	1.000		1.000		1.000	
**Husband/partner:**						
**Jealous if woman talks with other men**						
Yes			1.392	0.058	1.417	0.110
No (rc)			1.000		1.000	
**Accuses woman of unfaithfulness**						
Yes			2.431	**0.000**	2.233	**0.000**
No (rc)			1.000		1.000	
**Doesn’t permit her to meet female friends**						
Yes			1.254	0.224	1.592	**0.036**
No (rc)			1.000		1.000	
**Limits her contact with family**						
Yes			1.299	0.245	1.052	0.831
No (rc)			1.000		1.000	
**Insists on knowing where she is**						
Yes			2.003	**0.000**	1.532	**0.036**
No (rc)			1.000		1.000	
**Residence**						
Rural					0.906	0.709
Urban (rc)					1.000	
**Women’s education level**						
None (rc)					1.000	
Primary					1.228	0.401
Secondary or higher					1.299	0.437
**Woman owns a house alone or jointly**						
Yes					1.227	0.333
No (rc)					1.000	
**Women’s occupation**						
Not working (rc)					1.000	
Professional					0.098	**0.000**
Agriculture					0.803	0.306
Sales					0.815	0.451
**Wealth index**						
Poorest (rc)					1.000	
Poorer					0.711	0.194
Middle					0.880	0.661
Richer					0.489	**0.026**
Richest					0.452	**0.027**
**Region**						
Central (rc)					1.000	
Eastern					1.413	0.187
Northern					1.708	0.072
Western					1.549	0.111
**Parity**						
None (rc)					1.000	
1-4 children					3.603	**0.001**
5+ children					4.631	**0.000**
**Woman afraid of partner**						
Never (rc)					1.000	
Sometimes					3.169	**0.000**
Often					3.067	**0.000**
**Frequency of getting drunk**						
Never (rc)					1.000	
Sometimes					3.012	**0.000**
Often					1.670	**0.010**
**Respondent’s father ever beat her mother**						
Yes					2.163	**0.000**
No (rc)					1.000	

Bold means p-value <0.05.

Most measures of male partners’ behaviour as reported by women were significantly associated with IPPV. Women whose partners: accused them of unfaithfulness (OR = 2.23; p < 0.001); did not permit them to meet their female friends (OR = 1.59; p = 0.04) and insisted on knowing where they were (OR = 1.53; p = 0.04) were more likely to experience IPPV within 12 months prior to the survey.

Women’s attitude towards their partner, in terms of whether women were afraid of their partners was significantly associated with IPPV. Women, who were sometimes afraid of their partners (OR = 3.17; p < 0.001) and those who were often afraid of their partners (OR = 3.07; p <0.001) had increased likelihood of experiencing IPPV within 12 months prior to the survey, compared to those who were never afraid.

Partner’s excessive alcohol consumption was significantly associated with IPPV. Women whose partners sometimes (OR = 3.01; p < 0.001) or often (OR = 1.67; p = 0.01) got drunk with alcohol were more likely to report IPPV than those whose partners never got drunk.

One of the economic empowerment indicators – women’s occupation, was significantly associated with IPPV. Women in professional employment had decreased odds of reporting IPPV compared to non-working women (OR = 0.09; p < 0.001).

Among the socio-demographic factors considered, only wealth index and parity were significantly associated with IPPV. Women from richer (OR = 0.49; p = 0.03) and richest (OR = 0.45; p = 0.03) households had decreased odds of experiencing IPPV within 12 months prior to the survey, compared to those from the poorest households. Women who had 1–4 children (OR = 3.6; p = 0.001) and 5 or more children (OR = 4.63; p < 0.001) were more likely to experience IPPV compared to those who had no children.

As part of the contextual factors, model 3 included a history of women (respondents) witnessing parental physical violence. Women who witnessed their fathers beat their mothers were more likely (OR = 2.16; p < 0.001) to be beaten by their partners compared to those who had not witnessed such violence.

## Discussion

The prevalence of IPPV among women in union in Uganda remains relatively high (41%), although there is a slight decline from 48% in 2006 [7]. This prevalence level is still high compared to elsewhere in the sub-region, for instance Tanzania [29] and other developing countries [23,24,30-32].

Male partners’ behaviours, including alcohol consumption, controlling tendencies, and fear of male partners by their wives/female partners were significantly associated with IPPV among women in union in Uganda. Such behaviours explain the significant association between women’s fear of their spouses/partners and IPPV. Women, whose partners accused them of unfaithfulness, did not permit their contact with female friends and insisted on knowing where they were, had increased odds of reporting IPPV than those whose partners were less controlling regarding these issues. This finding is in consonance with findings from other sub-Saharan African countries such as Mozambique [33], Nigeria [19], and developing countries like Vietnam [34], Nepal [35] (see also WHO multi-country studies) [23,24]. Male partner controlling behaviours are not only a precursor to violence but could also be evidence that women were already experiencing violence. Controlling behavioural tendencies in this case appear to be closely associated with suspicions of infidelity. Antai’s study of IPV among Nigerian women established that partners’ controlling behavioural tendencies were significantly associated with IPPV. Her analysis aggregates variables addressing controlling behaviour, decision-making and attitudes justifying physical violence [19]. We opted to consider individual rather than aggregated variables to facilitate identification of specific measures that significantly influence IPPV for purposes of targeted responses by practitioners. With regard to partner controlling behaviour, the results show that in the Ugandan context, accusing the female partner of infidelity is the strongest measure, followed by limiting her contact with female friends and insistence on knowing her whereabouts.

Similarly, fear of the male partner was significantly associated with IPPV and could be a precursor as well as a result of IPPV. Fear of the partner which could be a result of experiencing violence, disempowers the women and in turn aggravates the cycle of physical violence [36].

Frequent and excessive alcohol consumption by male partners was significantly associated with IPPV, particularly among women whose partners sometimes or often got drunk. The fact that about 40% of the partners got drunk is a major cause for concern (Table 2). Recent studies in Uganda using the 2006 UDHS data [31,32,37] reported a similar finding. Studies elsewhere in: South Africa [38], Ethiopia [22,39], Rwanda [30], Botswana [39], China [40], Nepal [35], Indonesia [41], Poland [42], USA [43] and WHO multi-country study [23] confirmed the relationship between alcohol drinking and intimate partner physical violence.

Economic empowerment in form of women’s occupation was significantly associated with IPPV, where women in professional employment had decreased odds of experiencing IPPV compared to those who were not working. Professional employment may not only entail access to better incomes and therefore improvement in wealth, but is also accompanied by exposure, and enhancement of social status, which can mitigate the risk of IPPV. The results show that increase in wealth at household level reduces the odds of experiencing IPPV. A WHO multi-country study reported that higher socio-economic status protected women from IPPV [23].

Some studies have asserted that economic empowerment is not the sole protector of women against IPPV [13]. For instance, better incomes and therefore, access and control over economic resources does not necessarily protect women from IPPV as the case of Mexico [9] and USA [44]. In this study, ownership of resources (house) and decision making concerning spending women’s incomes were not significantly associated with IPPV (see Table 3). Qualitative sources of an urban based study among business women revealed that business women of high economic status experienced IPPV more than their lower economic status counterparts [15]. The fact that professional employment reduces the risk of IPPV implies that incomes have to be combined with social status. It is also important to consider the socio-economic status of the spouse [14].

In contrast with other studies [19], agency in terms of decision making autonomy regarding health care, major purchases, daily expenditures and attitudes justifying physical violence and economic empowerment in terms of ownership of resources were not associated with IPPV both at bivariate (Table 1) in the final model of our study (Table 3). An index for decision making measures (and one for women’s attitudes) was developed and fitted with IPPV with no significant association. This is contrary to the findings of Antai’s study of IPV among Nigerian women which reported that women’s decision making autonomy was significantly associated with IPPV, where decision making autonomy reduced the risk of IPPV (see also Lamichhane et al. with reference to Nepal [45]. Our findings are also contrary to studies in the region and elsewhere that link decision making autonomy to increase in risk of IPPV [11,15,44]. This highlights the importance of analysis of the socio-economic context specific population under study.

Socio-demographic factors namely women’s wealth index, parity and witnessing of parental violence were associated with IPPV. Parity has been associated with increase in IPPV [46]. Our findings too show that the odds of experiencing IPPV among women in union increase with increase in number of children. A US facility based study also revealed that the odds of occurrence of IPPV increased with each additional pregnancy [47,48]. Increase in number of children implies divided attention due to childcare, and in some cases emotional and economic strain. It also coincides with advancement in age which often results in extra marital relations, among other conflicts, that can result in IPPV.

As established in Uganda [17] and elsewhere [20,22] witnessing of parental IPPV perpetrated by fathers on the part of the respondents, is significantly associated with reports of experience of IPPV. Such experiences become part of socialization that nurtures attitudes that tolerate or accept IPPV, which contributes to actual occurrence of IPPV. This leads to intergenerational transmission of IPPV [49].

## Conclusions

The analysis reveals that factors associated with IPPV in Uganda are mainly related to male partners’ behaviours specifically, control behaviours associated with suspicion of infidelity; and getting drunk. These factors explain the significance of women’s fear of their partners which could also be a consequence of IPPV. In such a context, women’s empowerment has limited mitigating effect on IPPV, reflected only in relation to professional employment. Whereas household wealth had a mitigating effect on IPPV, witnessing of parental IPPV and parity increased the likelihood of IPPV. It is apparent that factors associated with IPPV vary depending on the social context of the women and their partners.

Based on these findings, analyses of empowerment and IPPV should consider social contexts. We recommend qualitative inquiry into the socio-economic issues surrounding IPPV in Uganda. Addressing IPPV in Uganda requires concerted efforts that target men to address excessive alcohol consumption and raise awareness and instil security in relationships. Problem drinking and insecurities in relationships are indicators of disempowerment! Consciousness raising and programmes geared towards countering perpetuation and tolerance of violence in the domestic sphere should be promoted. Empowerment programs should not only address women but men as well.

## Competing interests

The authors declare that they have no competing interests.

## Authors’ contributions

BK and SOW conceived and designed the study. BK wrote the background and SOW analysed the data. Both SOW and BK were involved in the interpretation of the results and drafting of the manuscript. PN was involved in drafting and reviewing the manuscript. PN, AK, BK and SOW read and reviewed the manuscript. All authors read and approved the manuscript.

## Authors’ information

BK is a Lecturer and a former Chair of the Department of Population Studies, School of Statistics and Planning, College of Business and Management, Makerere University. BK holds a PhD in Sociology, Masters in Development Studies (Women and Development). Has research on health and gender issues. SOW is an Assistant Lecturer at the Department of Population Studies and also a PhD student at Makerere University. His research is focusing on “Disparities of Access to Healthcare among Older persons in Uganda”. He holds a Master of Science in Population and Reproductive Health and a Bachelor of Science in Population Studies. In addition, he has research interests in Sexual and Gender-Based Violence. PN is an Assistant Lecturer and Chair, Department of Population Studies, Makerere University. She holds a Master of Public Health from Lund University, Sweden and a Master of Science in Population and Reproductive Health (Makerere University) and a Bachelor of Science in Population Studies. Her research interests are in Child Health and Sexual and Gender-Based Violence. AK is an Assistant Lecturer at the Department of Population Studies, Makerere University. She holds a Master of Arts in Demography (Makerere University) and a Bachelor of Arts in Education. Her research interests lie in family planning, maternal and child health, fertility and gender studies.

## Pre-publication history

The pre-publication history for this paper can be accessed here:

http://www.biomedcentral.com/1471-2458/13/1112/prepub
